# Association of motor index scores with fall incidence among community-dwelling older people

**DOI:** 10.1186/s12877-022-03680-6

**Published:** 2022-12-30

**Authors:** Xiao Liu, Ayiguli Abudukeremu, Yuan Jiang, Zhengyu Cao, Maoxiong Wu, Kai Zheng, Jianyong Ma, Runlu Sun, Zhiteng Chen, Yangxin Chen, Yuling Zhang, Jingfeng Wang

**Affiliations:** 1 Department of Cardiology, Sun Yat-Sen Memorial Hospital of Sun Yat-Sen University, Guangzhou, China; 2 Guangdong Province Key Laboratory of Arrhythmia and Electrophysiology, Guangzhou, China; 3 Guangzhou Key Laboratory of Molecular Mechanism and Translation in Major Cardiovascular Disease, Sun Yat-Sen Memorial Hospital, Sun Yat-Sen University, Guangzhou, China; 4 Medical Care Strategic Customer Department, China Merchants Bank Shenzhen Branch, Shenzhen, China; 5 Department of Pharmacology and Systems Physiology University of Cincinnati College of Medicine, Cincinnati, USA

**Keywords:** Motor index, Fall, TILDA

## Abstract

**Background:**

Several kinds of motor dysfunction have been studied for predicting future fall risk in community-dwelling older individuals. However, no study has tested the ability of the fine motor index (FINEA) and gross motor index (GROSSA) to predict the risk of falling, as well as the specific fall type.

**Objective:**

We investigated the associations of FINEA/GROSSA scores with fall risk, explained falls, and unexplained falls.

**Methods:**

A total of 6267 community-dwelling adults aged ≥ 50 years from the Irish Longitudinal Study on Aging (TILDA) cohort were included. First, the associations of FINEA and GROSSA scores with the history of total falls, explained falls and unexplained falls were assessed in a cross-sectional study and further verified in a prospective cohort after 2 years of follow-up by Poisson regression analysis.

**Results:**

We found that high FINEA and GROSSA scores were positively associated with almost all fall histories (FINEA scores: total falls: adjusted prevalence ratio [aPR] = 1.28, *P* = 0.009; explained falls: aPR = 1.15, *P* = 0.231; unexplained falls: aPR = 1.88, *P* < 0.001; GROSSA scores: total falls: aPR = 1.39, *P* < 0.001; explained falls: aPR = 1.28, *P* = 0.012; unexplained falls: aPR = 2.18, *P* < 0.001) in a cross-sectional study. After 2 years of follow-up, high FINEA scores were associated with an increased incidence of total falls (adjusted rate ratio [aRR] = 1.42, *P* = 0.016) and explained falls (aRR = 1.51, *P* = 0.020) but not with unexplained falls (aRR = 1.41, *P* = 0.209). High GROSSA scores were associated with an increased incidence of unexplained falls (aRR = 1.57, *P* = 0.041) and were not associated with either total falls (aRR = 1.21, *P* = 0.129) or explained falls (aRR = 1.07, *P* = 0.656). Compared with individuals without limitations in either the FINEA or GROSSA, individuals with limitations in both indices had a higher risk of falls, including total falls (aRR = 1.35, *P* = 0.002), explained falls (aRR = 1.31, *P* = 0.033) and unexplained falls (aRR = 1.62, *P* = 0.004).

**Conclusion:**

FINEA scores were positively associated with accidental falls, while GROSSA scores were positively associated with unexplained falls. The group for whom both measures were impaired showed a significantly higher risk of both explained and unexplained falls. FINEA or GROSSA scores should be investigated further as possible tools to screen for and identify community-dwelling adults at high risk of falling.

**Supplementary Information:**

The online version contains supplementary material available at 10.1186/s12877-022-03680-6.

## Background

Falling is a prevalent geriatric syndrome, affecting approximately one-third of older persons per year [1]. Notably, falling is frequently associated with serious complications in older people. For example, more than 30% of falls require medical attention, and approximately 5–7% of falls result in a fracture, which contributes to high mortality among older adults [2–4].

Predictive factors for the risk of falls have been investigated for decades, and factors including visual deficits, muscle strength, motor function, and postural control have been associated with the risk of falls [5, 6]. Among these factors, motor function, gait and balance are the most studied and have been shown to be firmly linked to the risk of falls in older people. As one of the widely used standardized measures of motor function, activities of daily living (ADL) are based on the most basic independent activities involved in daily life, such as eating and dressing, and require normal motor functioning, gait and balance [7]. Hence, evaluating an individual’s ADL to primarily and secondarily prevent fall risk is important.

Motor skills are divided into fine motor and gross motor skills, which include smaller muscle movements such as eating and large muscle movements such as walking, respectively. As studies with infants/children have shown that fine and gross motor skills influence the development of other body skills and have different developmental paths, further analysis of these skills should be considered separately [8–10]. As previous studies have shown that fine motricity is more sensitive for the early stage of paratonia and that some specific gross motor skills involve fine motor adjustment regulation, investigating these skills in an appropriate combined way is critical [10, 11].

To reduce the risk of falls, relevant training has been widely explored. A meta-analysis that assessed the preventive role of exercise on fall risk among community‐dwelling individuals showed that functional and balance exercises could reduce the rate of falls by 24% [12]. Fall prevention trials (both multifactorial and single-factor interventions) showed significant effectiveness among cognitively normal older individuals, while effectiveness was not shown in those with cognitive impairment [13, 14]. Detecting the risk of falls in prevention trials with individuals with normal cognitive function would be more significant, indicating the general screening of fall risk in these populations. As multiple screening tools, including the Activities-specific Balance Confidence scale, Berg Balance scale, and Timed Up and Go test, have been developed for assessing fall risk but failed to achieve both high sensitivity and specificity, Park SH suggested using several assessment tools with distinctive characteristics to increase the overall predictive accuracy [15, 16]. However, with an enormous number of older people worldwide, both the sensitivity and cost of screening methods should be considered to maximize revenue [17]. Therefore, more sensitive and specific predicting tools that could be used alone or in combination with low-cost tools with significant improvement to previous tools should be further investigated.

The fine motor index (FINEA) and gross motor index (GROSSA) are measured by counting the ADL-specific functions that subjects fail to accomplish and were proposed based on the Irish Longitudinal Study on Aging (TILDA) cohort. The TILDA is a large prospective study investigating social, economic, and health factors in Irish community-dwelling older adults [18]. To date, limited studies have investigated the association of ADL with the risk of falls, and no study has attempted to further investigate the association of FINEA and GROSSA scores with the risk of falls [19, 20]. Hence, we were curious about the association between FINEA or GROSSA scores and the risk of falling.

Explained falls were considered to be caused by extrinsic (surrounding) risk factors, including poor lighting and poor footwear, while unexplained falls were considered to be caused by intrinsic (multiple medical conditions) risk factors, including muscle weakness, poor vision, and chronic diseases (cardiovascular or autonomic issues) [21]. It is therefore of vital importance to distinguish between the two types of falls for in-depth evaluation and earlier intervention. Hence, based on the TILDA cohort, we aimed to assess the associations between FINEA and GROSSA scores and the risk of falling in older people and further analyzed the explained and unexplained falls to identify a possible tool to screen for and identify community-dwelling adults at high risk of falling.

## Methods

### Study sample

We obtained a dataset from the TILDA. The study design, inclusion/exclusion criteria, and follow-up of the TILDA have been published previously [18]. Briefly, the TILDA is a national study of Irish adults aged 49 years or older that aimed to assess the impact of health, social and financial circumstances on the aging process in older Irish individuals. The TILDA includes three data collection waves: wave 1 (from October 2009 to July 2011), wave 2 (from February 2012 to March 2013), and wave 3 (from March 2014 to October 2015). Written informed consent was obtained from cohort participants, and the study protocols were approved by the appropriate institutional ethical review boards. The TILDA was conducted in accordance with the Declaration of Helsinki.

Considering that dementia is a syndrome of cognitive impairment that can affect memory and that the record of falls in the TILDA was obtained through a questionnaire, individuals with comorbid dementia (recorded by a self-reported doctor-diagnosed questionnaire) in wave 2 were excluded [22].

### FINEA and GROSSA assessments

The definition of the FINEA and GROSSA assessments has been described by our previous report and TILDA relevant documentation [18, 23]. The FINEA and GROSSA scores were measured by counting the specific functions that subjects failed to accomplish, which were simple and feasible to obtain by self-report questionnaires. The FINEA measures included 3 items: picking up a small coin from a table, eating (such as cutting up food), and dressing. The GROSSA measures included 5 items: walking 100 m, walking across a room, climbing one flight of stairs without resting for long periods, getting in or out of bed, bathing or showering. These items were summed, and a high index score represented decreased motor function.

### Covariables

Demographic, clinical, and comorbidity data were recorded. Cognitive functioning was assessed using the Mini-Mental State Examination score (0–30), and scores less than 24 were considered indicative of cognitive impairment. Educational levels were defined as primary, secondary, and high education. Self-reported smoking status was classified as never smoker, past smoker, or current smoker. Physical activity levels were divided into three groups using the short form eight-item version of the International Physical Activity Questionnaire as follows: low, moderate, or high physical activity levels. The baseline self-reported doctor-diagnosed diseases included cardiovascular disease, diabetes or high blood sugar, stroke, mini-stroke or transient ischemic attack (TIA), and eye diseases (glaucoma, cataracts, age‐related macular degeneration).

### Outcomes of falls

In wave 2 and wave 3, the participants were asked “Have you fallen in the last year?” and “Have you had any falls since the last interview?”, respectively. In both waves, the participants with fall histories or incident falls were further asked, “Were any of these falls nonaccidental, i.e., with no apparent or obvious reason?”. Unexplained and explained falls were defined according to the participants’ answers: participants who answered ‘Yes’ to this question were classified as having a history of unexplained falls. Individuals with a history of both unexplained and explained falls were included in the group of unexplained falls.

### Statistical analyses

The data were analyzed using PASS Version 15.0 and SPSS Statistics Version 25.0 (IBM SPSS Statistics, IBM Corporation, Chicago, IL, USA) for Windows and R Programming Language (version 3.5.1). Power was computed based on the proportion in our study. A two-sided Z-test was used in the computation, which was based on the FINEA and GROSSA categorical groups, while the 2 degrees of freedom chi-square test was used for the combined motor index [24, 25]. The significance level (alpha) of the test was 0.05. In a cross-sectional study, analysis based on FINEA and GROSSA categorical groups achieved 100% power to detect a difference between the group proportions of -0.1830 for total fall history. Further combined motor index analysis also achieved 100% power to detect an effect size of 0.1518. In a prospective cohort study, analysis based on FINEA and GROSSA categorical groups achieved 97.78% and 99.76% power to detect a difference between the group proportions of -0.1390 and -0.1230, respectively, for the risk of total falls. Further combined motor index analysis achieved 100% power to detect an effect size of 0.0953.

The normality of the data was analyzed using the Kolmogorov‒Smirnov (KS) test. Normally distributed continuous variables are expressed as the means with standard deviations (SDs), and nonnormally distributed variables are expressed as medians with interquartile ranges (IQRs). The differences in the continuous variables between the groups were compared using unpaired Student's t tests (normal distribution) or Wilcoxon-Mann‒Whitney tests (nonnormal distribution). The categorical variables, which are reported as counts and percentages, were compared using χ2 tests.

First, FINEA (scores of 0 to 3) and GROSSA (scores of 0 to 5) scores from wave 2 data were analyzed as ordinal variables. Then, we further combined FINEA and GROSSA scores, which included scores from 0 to 8. Second, both FINEA and GROSSA scores were categorized into binary variables (FINEA score = 0 and FINEA score = 1–3, GROSSA score = 0 and GROSSA score = 1–5) as we previously reported [23]. We then combined FINEA and GROSSA scores to create three groups: Group 1: FINEA score = 0 and GROSSA score = 0; Group 2: FINEA score = 1–3 and GROSSA score = 0 or FINEA score = 0 and GROSSA score = 1–5; and Group 3: FINEA score = 1–3 and GROSSA score = 1–5. These groups correspond to no limitations in either FINEA or GROSSA, one limitation in either FINEA or GROSSA, and limitations in both FINEA and GROSSA, respectively.

This study comprised two stages to interpret the association of FINEA or GROSSA scores with falls (total falls, explained falls, unexplained falls). First, a cross-sectional study was conducted using data from wave 2, allowing us to investigate whether there was an association between FINEA and GROSSA scores and fall history. Second, after excluding subjects with a history of falls in wave 2, we analyzed the data as a prospective cohort study where we assessed the motor indices measured at wave 2 with subsequent falls at the 2-year follow-up (Wave 3). As a rare disease assumption is violated, both univariate and multivariate Poisson regression with robust variance were used to analyze the dependent and independent variables and were expressed as prevalence ratios (PRs) and 95% confidence intervals (CIs) in the cross-sectional study and rate ratios (RRs) and 95% CIs in the prospective cohort study [26–28]. The adjusted variables were selected by univariate Poisson regression (*P* < 0.1) from all baseline factors and considered meaningful clinical variables (Table S1). Hence, the adjusted variables in the multivariable Poisson regression included age, sex, exercise, smoking, diabetes mellitus (DM) or high blood sugar, stroke, mini-stroke or transient ischemic attack (TIA), eye disease, history of fainting, fear of falling, cognitive impairment, and cardiovascular disease. For further sensitivity analysis, we excluded age, exercise, fear of falling, cognitive impairment and cardiovascular disease from the adjusted variables in Supplemental model 1 and included education level, arthritis, and history of hip fracture in the adjusted variables as Supplemental model 2 (Table S2). *P* < 0.05 was considered statistically significant in all analyses.

## Results

### Cross-sectional study

**Fig. 1:**
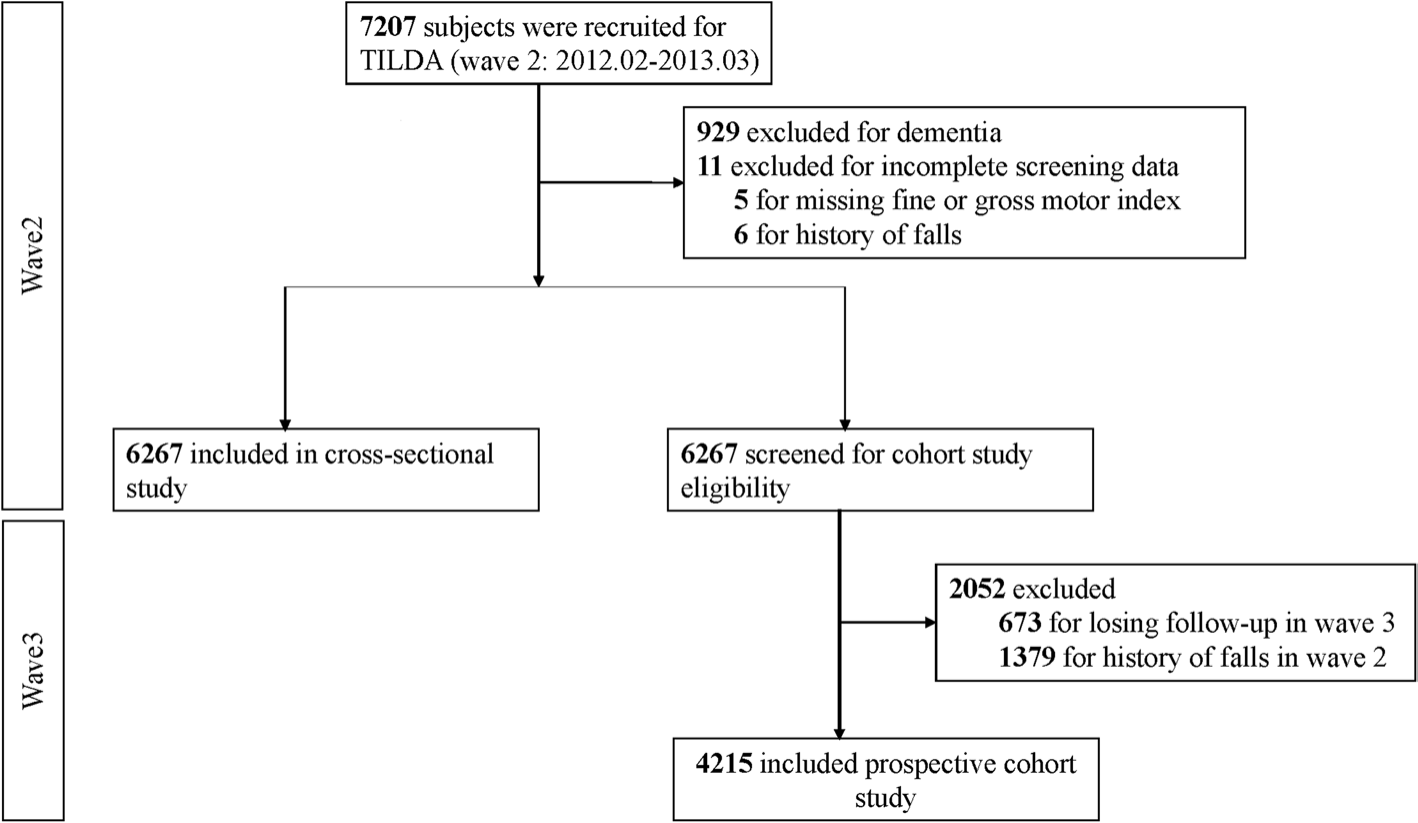
Participant flow in a cross-sectional study and prospective cohort study of the association between fine/gross motor index and risk of falls in participants included in TILDA. Note: TILDA: Irish Longitudinal Study on Aging

In total, 7,207 subjects were recruited for wave 2 of the TILDA. After excluding subjects with comorbid dementia and missing data (*N* = 929 for dementia; *N* = 5 for missing FINEA or GROSSA scores; *N* = 6 for missing data on the history of falls), 6,267 subjects were included. A flowchart of the selection of eligible individuals from the TILDA is shown in Fig. 1. The basic characteristics of the included subjects are presented in Table 1. Among these basic characteristics, data on body mass index, history of fainting and eye disease were collected from wave 1 due to the lack of relevant information in wave 2, and data for the other characteristics were collected from wave 2. The median age was 64.0 (57.0–72.0) years, 45% of the subjects were male, and 12.5% of the subjects had comorbid eye disease. More than half of the individuals had moderate or high self-reported physical activity levels (68.3%), and cardiovascular disease was the most common comorbidity. The median FINEA and GROSSA scores were 0 (0,0) and 0 (0,0), respectively. Notably, 22.0% of the individuals in our study reported a history of falls in the past year.

**Table 1:** Baseline characteristics of subjects included in the cross-sectional study

Variables	N% or median (IQR) (*N* = 6267)
Age (years)	64.0 (57.0,72.0)
Sex (male%)	2821 (45.0)
BMI (kg/m^2^)^a^	28.1 (25.3, 31.3)
**Education level, n (%)**	
Lower	1733 (27.7)
Secondary	3640 (58.1)
High	894 (14.3)
**Levels of physical activity, n (%)**	
Low	1989 (31.7)
Moderate	2213 (35.3)
High	2065 (33.0)
**Smoking, n (%)**	
Never	4591 (73.3)
Past	1383 (22.1)
Current	293 (4.7)
History of fainting, n (%)	1173 (18.7)
**Fall since last interview, n (%)**	
No	4888 (78.0)
Single fall	837 (13.4)
Multiple falls	532 (8.5)
Do not know or skipped due to routing patterns	10 (0.2)
Unexplained falls, n (%)	300 (4.8)
Afraid of falling, n (%)	1544 (24.6)
**Unsteadiness**^b^**, n (%)**	
Very steady	4847 (77.3)
Slightly steady	714 (11.4)
Slightly unsteady	574 (9.2)
Very unsteady	132 (2.1)
**Comorbidities, n (%)**	
Cognitive impairment	379 (6.0)
CVD	2531 (40.4)
DM or high blood sugar	457 (7.3)
Stroke	97 (1.5)
Mini stroke or TIA	134 (2.1)
Eye disease	782 (12.5)
Arthritis	436 (7%)
History of hip fracture	180 (2.9%)
FINEA	0 (0,0)
GROSSA	0 (0.0)

*CVD* Cardiovascular disease, *DM* Diabetes, *TIA* Transient Ischemic Attack, *FINEA* Fine motor index, *GROSSA* Gross motor index. Physical activity levels were divided into three groups using the short form eight-item version of the International Physical Activity Questionnaire as follows: low, moderate, or high. Cognitive functioning was assessed using the MMSE score (0–30) and less than 24 is considered indicative of cognitive impairment

^a^Values available in 4665 participants

^b^Self-reported unsteadiness during walking

### Associations between baseline FINEA/GROSSA scores and fall history

We first explored the association between FINEA scores and fall history (total falls, explained falls, and unexplained falls) in a cross-sectional study from Wave 2. FINEA scores, GROSSA scores and their combination were all significantly related to each form of fall history in the unadjusted model. As motor indices were analyzed as ordinal variables, we found that FINEA scores were positively associated with the history of falls categorized as total falls (aPR = 1.19, *P* = 0.004) and unexplained falls (aPR = 1.46, *P* < 0.001) but not with explained falls (aPR = 1.14, *P* = 0.116) after adjusting for age, sex, exercise, smoking, DM or high blood sugar, stroke, mini-stroke or TIA, eye disease, history of fainting, fear of falling, cognitive impairment, and cardiovascular disease. GROSSA scores were positively associated with all histories of falls categorized as total falls (aPR = 1.17, *P* < 0.001), explained falls (aPR = 1.26, *P* < 0.001) and unexplained falls (aPR = 1.35, *P* < 0.001) after adjustment. The combined motor index scores were positively associated with all histories of falls categorized as total falls (aPR = 1.13, *P* < 0.001), explained falls (aPR = 1.11, *P* = 0.001), and unexplained falls (aPR = 1.25, *P* < 0.001) after adjustment (Table 2).

**Table 2:** Poisson regression analysis of fine motor index, and gross motor index with fall history in cross-sectional study

Outcome	Fall history/Total	AIC	B	Unadjusted PR (95%CI)	*P* (*P for trend)	Adjusted PR (95%CI)	*P* (*P for trend)
FINEA							
Total falls							
FINEA = 0	1236/5885	6694.5	0.25	1 (ref.)		1 (ref.)	
FINEA = 1–3	143/382			1.78 (1.50, 2.12)	< 0.001	1.28 (1.07, 1.54)	0.009
Ordinal (0–3)	1379/6267	6693.6	0.17	1.49 (1.35, 1.66)	< 0.001*	1.19 (1.06, 1.34)	0.004*
Explained falls							
FINEA = 0	989/5638	5695.7	0.14	1 (ref.)		1 (ref.)	
FINEA = 1–3	85/324			1.50 (1.20, 1.87)	< 0.001	1.15 (0.91, 1.46)	0.231
Ordinal (0–3)	1074/5962	5694.7	0.13	1.36 (1.17, 1.58)	< 0.001*	1.14 (0.97, 1.33)	0.116*
Unexplained falls							
FINEA = 0	244/4893	2075.8	0.63	1 (ref.)		1 (ref.)	
FINEA = 1–3	56/295			3.81 (2.85, 5.09)	< 0.001	1.88 (1.37, 2.57)	< 0.001
Ordinal (0–3)	300/5188	2076.0	0.38	2.31 (1.96, 2.73)	< 0.001*	1.46 (1.22, 1.76)	< 0.001*
GROSSA							
Total falls							
GROSSA = 0	1089/5506	6684.8	0.33	1 (ref.)		1 (ref.)	
GROSSA = 1–5	290/761			1.93 (1.69, 2.19)	< 0.001	1.39 (1.19, 1.62)	< 0.001
Ordinal (0–5)	1379/6267	6678.7	0.16	1.34 (1.27, 1.41)	< 0.001*	1.17 (1.10, 1.25)	< 0.001*
Explained falls							
GROSSA = 0	896/5313	5690.9	0.24	1 (ref.)		1 (ref.)	
GROSSA = 1–5	178/649			1.63 (1.38,1.91)	< 0.001	1.28 (1.05, 1.54)	0.012
Ordinal (0–5)	1074/5962	5686.9	0.14	1.27 (1.19, 1.36)	< 0.001*	1.26 (1.06, 1.25)	< 0.001*
Unexplained falls							
GROSSA = 0	191/4608	2063.9	0.78	1 (ref.)		1 (ref.)	
GROSSA = 1–5	109/580			4.53 (3.58, 5.74)	< 0.001	2.18 (1.62, 2.93)	< 0.001
Ordinal (0–5)	300/5188	2061.5	0.30	1.77 (1.64, 1.92)	< 0.001*	1.35 (1.22, 1.49)	< 0.001*
FINEA + GROSSA							
Total falls							
Group 1	1036/5327	6682.5	0.24	1 (ref.)		1 (ref.)	
Group 2	253/737			1.77 (1.54, 2.03)	< 0.001	1.34 (1.14, 1.58)	< 0.001
Group 3	90/203			1.51 (1.36, 1.68)	< 0.001	1.24 (1.09, 1.40)	< 0.001
Ordinal (0–8)	1379/6267	6679.2	0.12	1.24 (1.20, 1.29)	< 0.001*	1.13 (1.07, 1.18)	< 0.001*
Explained falls							
Group 1	861/5152	5691.1	0.17	1 (ref.)		1 (ref.)	
Group 2	163/647			1.51 (1.28, 1.78)	< 0.001	1.24 (1.03, 1.51)	0.027
Group 3	50/163			1.35 (1.17, 1.56)	< 0.001	1.14 (0.97, 1.33)	0.110
Ordinal (0–8)	1074/5962	5687.8	0.10	1.19 (1.14, 1.26)	< 0.001*	1.11 (1.04, 1.18)	0.001*
Unexplained falls							
Group 1	173/4466	2056.8	0.59	1 (ref.)		1 (ref.)	
Group 2	89/573			4.01 (3.10, 5.18)	< 0.001	2.01 (1.46, 2.77)	< 0.001
Group 3	38/151			2.55 (2.14, 3.04)	< 0.001	1.76 (1.42, 2.18)	< 0.001
Ordinal (0–8)	300/5188	2060.0	0.22	1.51 (1.43, 1.60)	< 0.001*	1.25 (1.16, 1.34)	< 0.001*

Group1: FINEA &GROSSA = 0, Group2: FINEA = 0 &GROSSA = 1–5 OR FINEA = 1–3 &GROSSA = 0, Group3: FINEA = 1–3&GROSSA = 1–5. In subgroup analysis of explained falls, the individuals with history of unexplained falls were excluded from total individuals. In subgroup analysis of unexplained falls, the individuals with history of explained falls were excluded from total individuals.5 individuals were excluded from subgroup analysis because of unknown falling type. Adjusted PR: adjusted for age, sex, smoking, DM or high blood sugar, stroke, mini-stroke or TIA, eye disease, history of fainting, exercise, afraid of falling, cognitive impairment, CVD. *AIC* Akaike Information Criterion, *PR* Prevalence ratio, *95% CI* 95% confidence interval, *FINEA* Fine motor index, *GROSSA* Gross motor index, *CVD* Cardiovascular disease, *DM* Diabetes, *TIA* Transient Ischemic Attack

^*^Represents “*P* for trend” when FINEA, GROSSA and combined motor index analyzed as ordinal variables

After adjustment, as the motor indices were analyzed as categorical variables, we found that FINEA scores were positively associated with histories of falls categorized as total falls (aPR = 1.28, *P* = 0.009) and unexplained falls (aPR = 1.88, *P* < 0.001) but not with explained falls (aPR = 1.15, *P* = 0.231). GROSSA scores were positively associated with all histories of falls categorized as total falls (aPR = 1.39, *P* < 0.001), explained falls (aPR = 1.28, *P* = 0.012) and unexplained falls (aPR = 2.18, *P* < 0.001). The combined motor index scores were positively associated with the histories of falls categorized as total falls (aPR for Group 2 = 1.34, *P* < 0.001, Group 3 = 1.24, *P* < 0.001) and unexplained falls (aPR for Group 2 = 2.01, *P* < 0.001, Group 3 = 1.76, *P* < 0.001), but only the middle group had a higher history of explained falls than the lowest group (aPR for Group 2 = 1.24, *P* = 0.027, Group 3 = 1.14, *P* = 0.110) (Table 2).

### Prospective cohort study

Positive associations between FINEA/GROSSA/combined motor index scores and fall history were found in the cross-sectional study. To further determine their associations with falling, we examined whether the motor indices from wave 2 were associated with the falls reported in wave 3. We excluded individuals with a history of falls in wave 2 (*N* = 1379) and those who were lost to follow-up (*N* = 673), ultimately resulting in the inclusion of 4,215 subjects in the prospective study. The details of the subject selection are shown in Fig. 1. The basic characteristics of the included individuals are presented in Table 3.

**Table 3:** Baseline characteristics of subjects included in prospective cohort study

Variables	FINEA = 0 (*N* = 4035)	FINEA = 1–3 (*N* = 180)	*P* -value	GROSSA = 0 (*N* = 3885)	GROSSA = 1–5 (*N* = 330)	*P* -value
Age (years)	62.0 (56.0,70.0)	68.0 (60.8,77.3)	< 0.001	62.0 (56.0,70.0)	67.0 (59.0,77.0)	< 0.001
Sex (male%)	1887 (46.8)	86 (47.8)	0.849	1849 (47.6)	124 (37.6)	0.001
BMI (kg/m^2^)^a^	28.0 (25.3,31.1) (*N* = 3131)	28.8 (25.8,32.6) (*N* = 135)	0.059	27.9 (25.3,31.0) (*N* = 3029)	29.9 (26.6,33.5) (*N* = 237)	< 0.001
**Education level, n (%)**			0.02			< 0.001
Lower	1007 (25.0)	58 (32.2)		938 (24.1)	127 (38.5)	
Secondary	2430 (60.2)	106 (58.9)		2356 (60.6)	180 (54.5)	
High	598 (14.8)	16 (8.9)		591 (15.2)	23 (7.0)	
**Levels of physical activity, n (%)**			< 0.001			< 0.001
Low	1143 (28.3)	100 (55.6)		1044 (26.9)	199 (60.3)	
Moderate	1451 (36.0)	52 (28.9)		1413 (36.4)	90 (27.3)	
High	1441 (35.7)	28 (15.6)		1428 (36.8)	41 (12.4)	
**Smoking, n (%)**			< 0.001			< 0.001
Never	3117 (77.2)	68 (37.8)		3068 (79.0)	117 (35.5)	
Past	800 (19.8)	74 (41.1)		732 (18.8)	142 (43.0)	
Current	118 (2.9)	38 (21.1)		85 (2.2)	71 (21.5)	
History of fainting, n (%)	682 (16.9)	36 (20.0)	0.327	649 (16.7)	69 (20.9)	0.061
Fall since last interview, n (%)	694 (17.2)	56 (31.1)	< 0.001	660 (17.0)	90 (27.3)	< 0.001
Unexplained falls, n (%)	163 (4.0)	17 (9.4)	< 0.001	145 (3.7)	35 (10.6)	< 0.001
Afraid of falling, n (%)	726 (18.0)	73 (40.6)	< 0.001	644 (16.6)	155 (47.0)	< 0.001
**Unsteadiness#, n (%)**			< 0.001			< 0.001
Very steady	3417 (84.7)	85 (47.2)		3368 (86.7)	134 (40.6)	
Slightly steady	374 (9.3)	42 (23.3)		344 (8.9)	72 (21.8)	
Slightly unsteady	219 (5.4)	40 (22.2)		166 (4.3)	93 (28.2)	
Very unsteady	25 (0.6)	13 (7.2)		7 (0.2)	31 (9.4)	
**Comorbidities, n (%)**						
Cognitive impairment	150 (3.7)	11 (6.1)	0.150	131 (3.4)	30 (9.1)	< 0.001
CVD	1465 (36.3)	110 (61.1)	< 0.001	1378 (35.5)	197 (59.7)	< 0.001
DM or high blood sugar	253 (6.3)	24 (13.3)	< 0.001	228 (5.9)	49 (14.8)	< 0.001
Stroke	37 (0.9)	6 (3.3)	0.005	34 (0.9)	9 (2.7)	0.003
Mini stroke or TIA	66 (1.6)	10 (5.6)	< 0.001	65 (1.7)	11 (3.3)	0.050
Eye disease	414 (10.3)	36 (20.0)	< 0.001	374 (9.6)	76 (23.0)	< 0.001
Arthritis	274 (6.8)	21 (11.7)	0.012	265 (6.8)	30 (9.1)	0.121
History of hip fracture	92 (2.3)	4 (2.2)	0.959	85 (2.2)	11 (3.3)	0.181
FINEA	0 (0,0)	1 (1,1)	< 0.001	0 (0,0)	0 (0,0)	< 0.001
GROSSA	0 (0,0)	0 (0,1)	< 0.001	0 (0,0)	1 (1,2)	< 0.001

*BMI* Body mass index, *CVD* Cardiovascular disease, *DM* Diabetes, *TIA* Transient Ischemic Attack, *FINEA* Fine motor index, *GROSSA* Gross motor index. Physical activity levels were divided into three groups using the short form eight-item version of the International Physical Activity Questionnaire as follows: low, moderate, or high. Cognitive functioning was assessed using the MMSE score (0–30) and less than 24 is considered indicative of cognitive impairment

^a^Values available in 3264 participants

### Associations between the baseline FINEA/GROSSA/combined motor index scores and falls after two years of follow-up

After adjustment, as the motor indices were analyzed as ordinal variables, we found that FINEA scores were positively associated with total falls (aRR = 1.26, *P* = 0.038), marginally unassociated with explained falls (aRR = 1.29, *P* = 0.059) and not with unexplained falls (aRR = 1.30, *P* = 0.179). GROSSA scores were positively associated with unexplained falls (aRR = 1.24, *P* = 0.030) but not with total falls (aRR = 1.06, *P* = 0.401) and explained falls (aRR = 0.95, *P* = 0.619). The combined motor indices were positively associated with the risk of falls categorized as unexplained falls (aRR = 1.18, *P* = 0.023) but were not associated with total falls (aRR = 1.08, *P* = 0.118) or explained falls (aRR = 1.03, *P* = 0.634) (Table 4).

**Table 4:** Poisson regression analysis of fine motor index, gross motor index with risk of falls in prospective cohort study

Outcome	Fall events/Total	AIC	B	Unadjusted RR (95% CI)	*P* (*P for trend)	Adjusted RR (95% CI)	*P* (*P for trend)
FINEA							
Total falls							
FINEA = 0	694/4035	4013.1	0.35	1 (ref.)		1 (ref.)	
FINEA = 1–3	56/180			1.81(1.38, 2.37)	< 0.001	1.42(1.07, 1.90)	0.016
Ordinal (0–3)	750/4215	4014.6	0.23	1.49(1.22,1.82)	< 0.001*	1.26(1.01, 1.56)	0.038*
Explained falls							
FINEA = 0	523/3864	3308.5	0.41	1 (ref.)		1 (ref.)	
FINEA = 1–3	38/162			1.73(1.25, 2.41)	0.001	1.51(1.07, 2.15)	0.020
Ordinal (0–3)	561/4026	3310.2	0.25	1.43(1.12,1.84)	0.005*	1.29(0.99,1.68)	0.059*
Unexplained falls							
FINEA = 0	163/3504	1359.6	0.34	1 (ref.)		1 (ref.)	
FINEA = 1–3	17/141			2.59(1.57, 4.27)	< 0.001	1.41(0.83, 2.39)	0.209
Ordinal (0–3)	180/3645	1359.5	0.26	1.91(1.36, 2.69)	< 0.001*	1.30(0.89,1.90)	0.179*
GROSSA							
Total falls							
GROSSA = 0	660/3885	4016.2	0.19	1 (ref.)		1 (ref.)	
GROSSA = 1–5	90/330			1.61(1.29,2.00)	< 0.001	1.21(0.95, 1.56)	0.129
Ordinal (0–5)	750/4215	4017.8	0.06	1.24(1.10, 1.40)	< 0.001*	1.06(0.92,1.22)	0.401*
Explained falls							
GROSSA = 0	509/3734	3313.2	0.07	1 (ref.)		1 (ref.)	
GROSSA = 1–5	52/292			1.31(0.98, 1.74)	0.066	1.07(0.78, 1.47)	0.656
Ordinal (0–5)	561/4026	3313.1	-0.05	1.08(0.91,1.29)	0.376*	0.95(0.78,1.16)	0.619*
Unexplained falls							
GROSSA = 0	145/3370	1357.1	0.45	1 (ref.)		1 (ref.)	
GROSSA = 1–5	35/275			2.96(2.04, 4.28)	< 0.001	1.57(1.02, 2.43)	0.041
Ordinal (0–5)	180/3645	1357.0	0.21	1.62(1.38,1.89)	< 0.001*	1.24(1.02,1.51)	0.030*
FINEA + GROSSA							
Total falls							
Group 1	636/3783	4012.7	0.22	1 (ref.)		1 (ref.)	
Group 2	82/354			1.36 (1.09, 1.73)	0.006	1.07 (0.82, 1.38)	0.620
Group 3	32/78			1.56 (1.31, 1.87)	< 0.001	1.35 (1.11, 1.63)	0.002
Ordinal (0–8)	750/4215	4016.2	0.08	1.21(1.11, 1.31)	< 0.001*	1.08(0.98, 1.20)	0.118*
Explained falls							
Group 1	489/3636	3310.7	0.18	1 (ref.)		1 (ref.)	
Group 2	54/326			1.23 (0.93, 1.63)	0.146	1.04 (0.76, 1.43)	0.794
Group 3	18/64			1.45 (1.14, 1.83)	0.002	1.31 (1.02, 1.68)	0.033
Ordinal (0–8)	561/4026	3313.2	0.03	1.13(1.00,1.27)	0.054*	1.03(0.90, 1.19)	0.634*
Unexplained falls							
Group 1	141/3288	1356.3	0.36	1 (ref.)		1 (ref.)	
Group 2	26/298			2.03 (1.34, 3.09)	< 0.001	1.07 (0.66, 1.74)	0.772
Group 3	13/59			2.27 (1.71, 3.01)	< 0.001	1.62 (1.17, 2.23)	0.004
Ordinal (0–8)	180/3645	1356.9	0.16	1.43(1.27,1.59)	< 0.001*	1.18(1.02,1.36)	0.023*

Group1: FINEA &GROSSA = 0, Group2: FINEA = 0 &GROSSA = 1–5 OR FINEA = 1–3 &GROSSA = 0, Group3: FINEA = 1–3&GROSSA = 1–5. In subgroup analysis of explained falls, the individuals with incident of unexplained falls were excluded from total individuals. In subgroup analysis of unexplained falls, the individuals with incident of explained falls were excluded from total individuals. Adjusted PR: adjusted for age, sex, smoking, DM or high blood sugar, stroke, mini-stroke or TIA, eye disease, history of fainting, exercise, afraid of falling, cognitive impairment, CVD. *AIC* Akaike Information Criterion, *RR* Rate ratio, *95% CI* 95% Confidence interval, *FINEA* Fine motor index, *GROSSA* Gross motor index, *CVD* Cardiovascular disease, *DM* Diabetes, *TIA* Transient Ischemic Attack

^*^Represents “*P* for trend” when FINEA, GROSSA and combined motor index analyzed as ordinal variables

After adjustment, as the motor indices were analyzed as categorical variables, we found that FINEA scores were positively associated with total falls (aRR = 1.42, *P* = 0.016) and explained falls (aRR = 1.51, *P* = 0.020) but not with unexplained falls (aRR = 1.41, *P* = 0.209). GROSSA scores were positively associated with unexplained falls (aRR = 1.57, *P* = 0.041) and unassociated with both total falls (aRR = 1.21, *P* = 0.129) and explained falls (aRR = 1.07, *P* = 0.656). The highest combined motor indices were positively associated with the risk of all types of falls, including total falls (aRR for Group 2 = 1.07, *P* = 0.620, Group 3 = 1.35, *P* = 0.002), explained falls (aRR for Group 2 = 1.04, *P* = 0.794, Group 3 = 1.31, *P* = 0.033) and unexplained falls (aRR for Group 2 = 1.07, *P* = 0.772, Group 3 = 1.62, *P* = 0.004) (Table 4).

### Sensitivity analysis

As age, exercise, fear of falling, cognitive impairment and cardiovascular disease were excluded from the adjusted variables, the positive association between FINEA scores and unexplained falls (aRR = 1.91, *P* = 0.016) and GROSSA scores and total falls (aRR = 1.43, *P* = 0.004) became significant. As education level, arthritis and history of hip fracture were added to the adjusted variables, the results were similar to those of the fully adjusted model (Table S2).

## Discussion

Among community-dwelling older people, our cross-sectional study showed that almost all FINEA, GROSSA and combined motor indices were positively associated with fall history. To further investigate the association of the motor index with the risk of falls, we performed a prospective cohort study. Our prospective cohort analysis showed that FINEA scores were positively associated with explained falls and that GROSSA scores were positively associated with unexplained falls. Individuals with simultaneously impaired FINEA and GROSSA scores could have a higher risk of falls, including total, explained and unexplained falls. Moreover, as shown in Table 4, a higher B value of Poisson regression analysis of categorical variables compared to ordinal variables indicated that the categorical motor index may be a better predictive tool for the prediction of future falls.

Explained and unexplained falls were caused by extrinsic and intrinsic risk factors. Fine skill abnormalities may be an earlier stage of motor dysfunction, and gross skill abnormalities may indicate more significant central abnormalities [10, 23, 29]. Hence, individuals may attribute the reason for falls to extrinsic risk factors, which might be caused by earlier states of body dysfunction. Generally, unexplained falls are associated with more intracranial injury and are more likely due to syncope or underlying chronic disease [30]. One-third of patients admitted to an orthopedic ward had unexplained falls [31]. Therefore, identifying individuals with both explained and unexplained falls is important. Further combinations of the FINEA and GROSSA indices showed a positive association with both explained and unexplained falls, indicating the expected predictive role. However, as the brain remains incompletely understood, the potential for diverse associations between different motor skills and fall types should be further studied.

To verify the positive association of the motor index with the risk of falls, we adjusted for relevant risk factors for fall incidents and found that the association of FINEA and GROSSA scores with fall risk was still significant. As multiple studies showed that education level, arthritis, and history of fracture were also associated with the risk of falls, we further adjusted for these risk factors in Supplemental model 2 (Table S2) in our Poisson regression analysis and found that the results were still significant, which further indicated that both FINEA and GROSSA scores may be independent predictors of the risk of falls [32–40]. In our sensitivity analysis, we also demonstrated that age, exercise, fear of fall, cognitive impairment and cardiovascular disease may modify the association between FINEA/GROSSA scores and the risk of falls.

Our study showed that FINEA, GROSSA and combined motor indices could predict the risk of falls. As both the FINEA and GROSSA are simple and feasible self-report questionnaires, they might be potential effective tools to screen for and identify community-dwelling older people who are at a high risk of falling. Future studies should investigate their sensitivity, specificity and related measures to determine their feasibility as screening tools. At the same time, further studies should compare the predictive abilities of the FINEA and GROSSA compared to existing tools for screening healthy older adults.

### Strengths and limitations

Our study has several strengths. We first introduced FINEA or GROSSA scores as being associated with the risk of falls in community-dwelling adults and conducted a subgroup analysis regarding data on explained and unexplained falls. This association persisted when adjusting for age, sex, cardiovascular disease, and cognitive impairment, which suggests a role of FINEA/GROSSA scores in falls in these community-dwelling participants. Second, this research was based on a well-designed study with a large sample size, which makes the results more credible. Third, both the FINEA and GROSSA are simple, feasible, self-report questionnaires that could be used as potential tools to screen for and identify older patients at high risk of falling.

Our study also has limitations. First, wave 2 of the TILDA did not collect information on gait alterations (such as speed or stability), which have been well established as having an association with falls [5, 41–43]. Second, previous studies have also shown that depression and antidepressant use were independently associated with falls [44]; however, wave 2 of the TILDA also did not include information on depression diagnosis and antidepressant use. Third, in our cross-sectional analysis, the association of the motor index with the presence of falls in the last year was studied, which cannot be considered completely cross-sectional, so the results should be further confirmed. Fourth, as both fall incidents and fall types rely on self-reports for 1 to 2 years, inaccuracies and memory bias could affect the accuracy of the study [45]. Fifth, as our investigation is an observational study, we cannot prove reliable cause-effect associations between the motor index and risk of falls, so randomized controlled trials should be further performed. Sixth, it should be clearly stated that our sample was Irish, so further analyses based on other populations, including Americans and Asians, should be further performed.

## Conclusion

Both FINEA and GROSSA scores were positively associated with total fall history among the Irish older population in a cross-sectional study. Further prospective cohort studies showed that FINEA scores were positively associated with accidental falls, while GROSSA scores were positively associated with unexplained falls based on self-reported recall. Individuals with simultaneously impaired FINEA and GROSSA scores could have a higher risk of falls, including total, explained and unexplained falls. Hence, the FINEA or GROSSA may be investigated further as possible tools to screen for and identify community-dwelling adults at high risk of falling. Patients with dysfunction of the FINEA or GROSSA may be an appropriate target for fall prevention interventions.

## Supplementary Information

**Additional file 1:** **Table S1.** Univariable Poisson regression analysis of fine motor index, gross motor index with risk of total falls in both of cross-sectional and prospective cohort study. **Table S2.** Poisson regression analysis of fine motor index, gross motor index with risk of falls in prospective cohort study.

## Data Availability

The datasets used and analyzed during the current study are available from the corresponding author on reasonable request.

## Abbreviations

aPR: Adjusted prevalence ratio
aRR: Adjusted rate ratio
CIs: Confidence intervals
DM: Diabetes mellitus
FINEA: Fine motor index
GROSSA: Gross motor index
IQRs: Interquartile ranges
KS: Kolmogorov‒Smirnov
SD: Standard deviation
TIA: Transient ischemic attack
TILDA: The Irish Longitudinal Study on Aging
