# Supplementary material for: Association of motor index scores with fall incidence among community-dwelling older people
Source: BMC Geriatr. 2022 Dec 30;22:1008. doi: 10.1186/s12877-022-03680-6 (PMC9805168; doi:10.1186/s12877-022-03680-6)
Supplement: Supplementary file 1 — Additional file 1: Table S1. Univariable Poisson regression analysis of fine motor index, gross motor index with risk of total falls in both of cross-sectional and prospective cohort study. Table S2. Poisson regression analysis of fine motor index, gross motor index with risk of falls in prospective cohort study. [file 12877_2022_3680_MOESM1_ESM.docx]

| **Variables** | **cross-sectional study**  **Unadjusted PR (95%CI)** | ***P*** | **prospective cohort study**  **Unadjusted RR (95% CI)** | ***P*** |
| --- | --- | --- | --- | --- |
| Age (years) | 1.02 (1.02, 1.03) | <0.001 | 1.03 (1.02, 1.04) | <0.001 |
| Sex | 1.26 (1.13, 1.40) | <0.001 | 1.32 (1.14, 1.53) | <0.001 |
| BMI (kg/m^2^) * | 1.01 (0.99, 1.02) | 0.379 | 1.00 (0.99, 1.02) | 0.693 |
| Education level | 1.03 (0.99,1.02) | 0.480 | 0.86 (0.77, 0.97) | 0.012 |
| Levels of physical activity | 0.91 (0.85, 0.97) | 0.006 | 0.90 (0.83, 0.99) | 0.025 |
| Smoking | 1.16 (1.06, 1.27) | <0.001 | 1.10 (0.96, 1.25) | 0.166 |
| History of fainting | 1.45 (1.29, 1.64) | <0.001 | 1.09 (0.90,1.31) | 0.369 |
| Afraid of falling | 2.21 (1.99, 2.46) | <0.001 | 1.66 (1.42, 1.95) | <0.001 |
| Unsteadiness^#^ | 1.47 (1.39, 1.56) | <0.001 | 1.45 (1.32, 1.59) | <0.001 |
| Cognitive impairment | 1.47 (1.22, 1.77) | <0.001 | 1.23 (0.88, 1.73) | 0.227 |
| CVD | 1.27 (1.14, 1.41) | <0.001 | 1.21 (1.05, 1.40) | 0.009 |
| DM or high blood sugar | 1.27 (1.05, 1.52) | 0.012 | 1.28 ((0.99, 1.66) | 0.062 |
| Stroke | 1.85 (1.35, 2.55) | <0.001 | 1.31 (0.70, 2.45) | 0.395 |
| Mini stroke or TIA | 1.23 (0.88, 1.71) | 0.226 | 1.57 (1.02, 2.42) | 0.042 |
| Eye disease | 1.40 (1.22, 1.62) | <0.001 | 1.56 (1.28, 1.90) | <0.001 |
| Arthritis | 1.03 (0.84, 1.27) | 0.746 | 1.41 (1.11, 1.80) | 0.005 |
| History of hip fracture | 1.57 (1.21, 2.02) | <0.001 | 1.30 (0.85, 1.98) | 0.230 |
| FINEA (0-3) | 1.49 (1.34, 1.66) | <0.001 | 1.49 (1.22, 1.82) | <0.001 |
| GROSSA (0-5) | 1.34 (1.27, 1.41) | <0.001 | 1.24 (1.10,1.40) | <0.001 |

**Table S1: Univariable Poisson regression analysis of fine motor index, gross motor index with risk of total falls in both of cross-sectional and prospective cohort study.**

BMI: body mass index, CVD: cardiovascular disease, DM: diabetes, TIA: Transient Ischemic Attack, FINEA: fine motor index, GROSSA: gross motor index. Physical activity levels were divided into three groups using the short form eight-item version of the International Physical Activity Questionnaire as follows: low, moderate, or high. Cognitive functioning was assessed using the MMSE score (0-30) and less than 24 is considered indicative of cognitive impairment.

^*^Values available in 4665 participants in cross sectional study part and 3266 participants in prospective study part, ^#^Self-reported unsteadiness during walking.

**Table S2. Poisson regression analysis of fine motor index, gross motor index with risk of falls in prospective cohort study.**

| **Outcome** | **Fall history/Total** | **Model1**  **RR (95% CI)** | ***P***  **(**P* for trend)** | **Model2**  **RR (95% CI)** | ***P***  **(**P* for trend)** |
| --- | --- | --- | --- | --- | --- |
| **FINEA** | | | | | |
| **Total falls** | | | | | |
| **FINEA=0**  **FINEA=1-3** | 694/4035  56/180 | 1 (ref.)  1.65 (1.24, 2.20) | <0.001 | 1 (ref.)  1.42 (1.06, 1.90) | 0.017 |
| **Ordinal (0-3)** | 750/4215 | 1.40 (1.14, 1.73) | 0.002***** | 1.25 (1.01, 1.55) | 0.040***** |
| **Explained falls** | | | | | |
| **FINEA=0**  **FINEA=1-3** | 523/3864  38/162 | 1 (ref.)  1.69 (1.20,2.38) | 0.003 | 1 (ref.)  1.52 (1.07, 2.15) | 0.019 |
| **Ordinal (0-3)** | 561/4026 | 1.40 (1.08, 1.82) | 0.010***** | 1.29 (0.99, 1.68) | 0.059***** |
| **Unexplained falls** | | | | | |
| **FINEA=0**  **FINEA=1-3** | 163/3504  17/141 | 1 (ref.)  1.91 (1.13, 3.22) | 0.016 | 1 (ref.)  1.39 (0.82, 2.37) | 0.226 |
| **Ordinal (0-3)** | 180/3645 | 1.63 (1.13, 2.35) | 0.009***** | 1.29 (0.88, 1.89) | 0.189***** |
| **GROSSA** | | | | | |
| **Total falls** | | | | | |
| **GROSSA=0**  **GROSSA=1-5** | 660/3885  90/330 | 1 (ref.)  1.43 (1.12, 1.82) | 0.004 | 1 (ref.)  1.21 (0.94, 1.56) | 0.132 |
| **Ordinal (0-5)** | 750/4215 | 1.17 (1.02, 1.33) | 0.022***** | 1.06 (0.92, 1.22) | 0.407***** |
| **Explained falls** | | | | | |
| **GROSSA=0**  **GROSSA=1-5** | 509/3734  52/292 | 1 (ref.)  1.20 (0.89, 1.64) | 0.234 | 1 (ref.)  1.08 (0.79, 1.48) | 0.627 |
| **Ordinal (0-5)** | 561/4026 | 1.03 (0.85, 1.24) | 0.777***** | 0.95 (0.78, 1.16) | 0.642***** |
| **Unexplained falls** | | | | | |
| **GROSSA=0**  **GROSSA=1-5** | 145/3370  35/275 | 1 (ref.)  2.16 (1.42, 3.28) | <0.001 | 1 (ref.)  1.55 (1.00, 2.39) | 0.050 |
| **Ordinal (0-5)** | 180/3645 | 1.44 (1.20, 1.72) | <0.001***** | 1.23 (1.01, 1.50) | 0.036***** |
| **FINEA+GROSSA** | | | | | |
| **Total falls** | | | | | |
| **Group 1**  **Group 2**  **Group 3** | 636/3783  82/354  32/78 | 1 (ref.)  1.26 (0.98, 1.62)  1.49 (1.24, 1.79) | 0.075  <0.001 | 1 (ref.)  1.07 (0.82, 1.38)  1.34 (1.11, 1.62) | 0.626  0.003 |
| **Ordinal (0-8)** | 750/4212 | 1.16 (1.06, 1.27) | 0.002***** | 1.08 (0.98, 1.20) | 0.122***** |
| **Explained falls** | | | | | |
| **Group 1**  **Group 2**  **Group 3** | 489/3636  54/326  18/64 | 1 (ref.)  1.17 (0.86, 1.58)  1.41 (1.11, 1.80) | 0.327  0.005 | 1 (ref.)  1.05 (0.76, 1.43)  1.32 (1.03, 1.69) | 0.779  0.031 |
| **Ordinal (0-8)** | 561/4026 | 1.10 (0.96, 1.25) | 0.168***** | 1.04 (0.90, 1.19) | 0.613***** |
| **Unexplained falls** | | | | | |
| **Group 1**  **Group 2**  **Group 3** | 141/3288  26/298  13/59 | 1 (ref.)  1.53 (0.96, 2.44)  1.97 (1.45, 2.68) | 0.075  <0.001 | 1 (ref.)  1.05 (0.65, 1.70)  1.60 (1.16, 2.21) | 0.842  0.004 |
| **Ordinal (0-8)** | 180/3645 | 1.32 (1.16, 1.50) | <0.001***** | 1.17 (1.02, 1.35) | 0.028***** |

Group1: FINEA &GROSSA=0, Group2: FINEA=0 &GROSSA=1-5 OR FINEA=1-3 &GROSSA=0, Group3: FINEA=1-3&GROSSA=1-5. In subgroup analysis of explained falls, the individuals with history of unexplained falls were excluded from total individuals. In subgroup analysis of unexplained falls, the individuals with history of explained falls were excluded from total individuals.5 individuals were excluded from subgroup analysis because of unknown falling type. Model 1: adjusted for sex, smoking, DM or high blood sugar, stroke, mini-stroke or TIA, eye disease, history of fainting, Model2: age, sex, smoking, DM or high blood sugar, stroke, mini-stroke or TIA, eye disease, history of fainting, exercise, afraid of falling, cognitive impairment, CVD, educational level, arthritis, history of hip fracture. RR: rate ratio, 95% CI: 95% confidence interval, FINEA: fine motor index, GROSSA: gross motor index, CVD: cardiovascular disease, DM: diabetes, TIA: Transient Ischemic Attack.

***** Represents “*P* for trend” when FINEA, GROSSA and combined motor index analyzed as ordinal variables.
